# Supplementary material for: Pathways Activated by Infected and Bystander Chondrocytes in Response to Ross River Virus Infection
Source: Viruses. 2022 Dec 31;15(1):136. doi: 10.3390/v15010136 (PMC9864161; doi:10.3390/v15010136)
Supplement: Supplementary file 1 [file viruses-15-00136-s001.zip › viruses-2102683-supplementary.pdf]

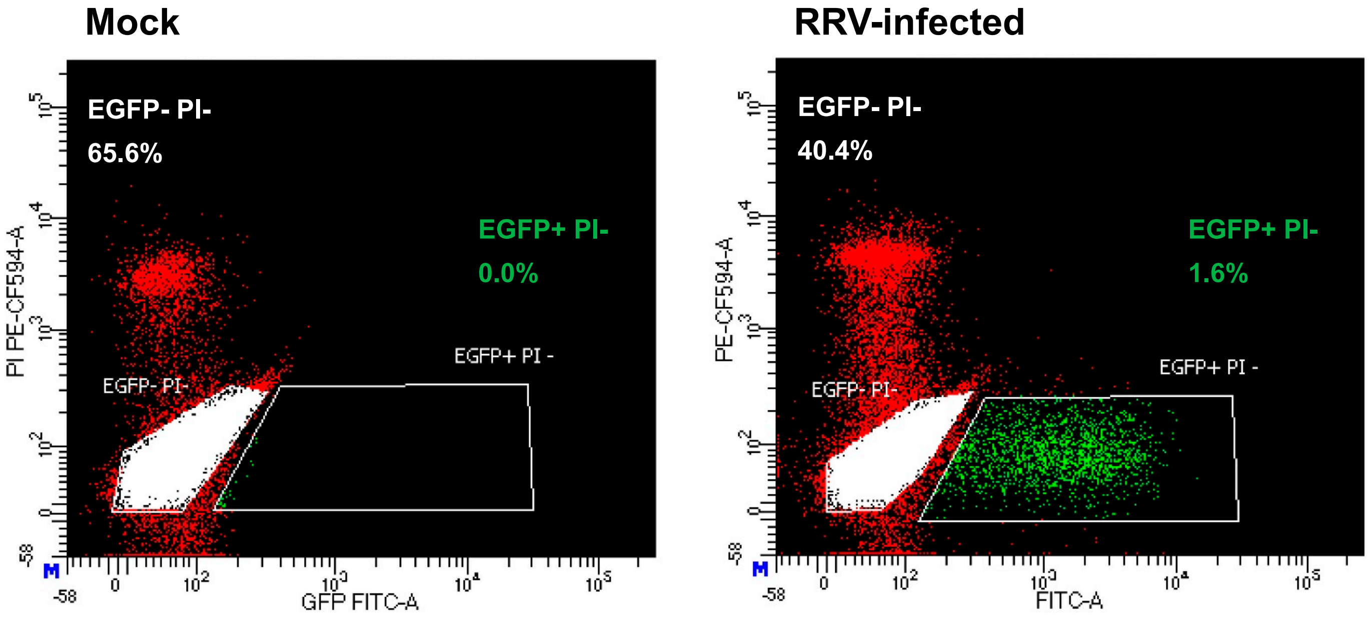

Figure S1. Cell sorting of mock- and RRV-infected chondrocyte cells. Chondrocytes were infected with RRV-GFP at MOI 10 for two days. The harvested cells were stained with PI and FSC/SSC-gated to remove cellular debris. Subsequently, PI-negative cells were sorted to select for viable cells, and GFP-positive (RRV-infected) and GFP-negative (bystander) cells sorted for downstream analysis. Mock-infected cells were used as controls (EGFP- PI-).
